# Nanostructuring of Additively Manufactured 316L Stainless Steel Using High-Pressure Torsion Technique: An X-ray Line Profile Analysis Study

**DOI:** 10.3390/ma17020454

**Published:** 2024-01-18

**Authors:** Jenő Gubicza, Kamilla Mukhtarova, Megumi Kawasaki

**Affiliations:** 1 Department of Materials Physics, Eötvös Loránd University, 1117 Budapest, Hungary; kamilla551@gmail.com; 2 School of Mechanical, Industrial and Manufacturing Engineering, Oregon State University, Corvallis, OR 97331-6001, USA; megumi.kawasaki@oregonstate.edu

**Keywords:** 316L steel, additive manufacturing, high-pressure torsion, X-ray line profile analysis, dislocation density, crystallite size, twin fault probability

## Abstract

Experiments were conducted to reveal the nanostructure evolution in additively manufactured (AMed) 316L stainless steel due to severe plastic deformation (SPD). SPD-processing was carried out using the high-pressure torsion (HPT) technique. HPT was performed on four different states of 316L: the as-built material and specimens heat-treated at 400, 800 and 1100 °C after AM-processing. The motivation for the extension of this research to the annealed states is that heat treatment is a usual step after 3D printing in order to reduce the internal stresses formed during AM-processing. The nanostructure was studied by X-ray line profile analysis (XLPA), which was completed by crystallographic texture measurements. It was found that the as-built 316L sample contained a considerable density of dislocations (10^15^ m^−2^), which decreased to about half the original density due to the heat treatments at 800 and 1100 °C. The hardness varied accordingly during annealing. Despite this difference caused by annealing, HPT processing led to a similar evolution of the microstructure by increasing the strain for the samples with and without annealing. The saturation values of the crystallite size, dislocation density and twin fault probability were about 20 nm, 3 × 10^16^ m^−2^ and 3%, respectively, while the maximum achievable hardness was ~6000 MPa. The initial <100> and <110> textures for the as-built and the annealed samples were changed to <111> due to HPT processing.

## 1. Introduction

Additive manufacturing (AM), also known as 3D printing, is a process in which objects are built up layer by layer [1,2]. The first step is to use a computer-aided design software to create a 3D solid model, which is then converted to a standard file format and sent to the AM machine. The part is then built layer by layer on the 3D printer, and the final step is to clean and finish the model [2]. Interest in AM processes has grown steadily in recent years [3]. This growing interest is linked to the advantages of AM compared to conventional manufacturing methods.

AM can be used in a wide range of industries, including aerospace [4,5,6], medical [7,8,9] and automotive [10,11] industries, due to its ability to create complex components and parts [12,13,14,15,16]. Furthermore, AM provides additional benefits, such as reduced material waste [1,12], the quick production of prototypes [14,15], a reduced cost for small production runs [12,17], environmental friendliness [18], and supply chain flexibility [19,20]. A large variety of materials, ranging from metals to polymers and ceramics, can be used to produce components using AM methods [21,22].

The most frequently employed AM techniques, as specified by the ASTM 52900 guidelines [23], encompass powder bed fusion, material extrusion, vat polymerization, direct energy deposition, binder jetting, material jetting and sheet lamination. The most common technique for the AM processing of metal and alloys is the powder bed fusion (PBF) process, which utilizes a laser or electron beam to melt and solidify layers of the powder to create the desired shape of the object. The most important parameters of PBF are hatch spacing, layer thickness, laser power, and laser scan speed [24]. Material extrusion utilizes a hot printhead, in which the thermoplastic filaments are melted and extruded through the nozzle, constructing the component in a layer-by-layer pattern [25]. Vat polymerisation, also known as stereolithography, employs an ultraviolet (UV) laser beam to scan and solidify the surface of a liquid monomer, resulting in the formation of a solid polymer [26].

The direct energy deposition (DED) process utilizes wire or metal powder flow as a feedstock material, which is melted by a laser or electron beam. Unlike powder bed fusion techniques, DED processes are not used to melt a material that is pre-laid in a powder bed but are used to melt materials as they are deposited [27]. Binder jetting (BJ) uses single or multiple nozzles to deliver a liquid binder to the surface of the powder bed, binding the powder particles together. The nozzle moves according to the predetermined path until a thin subsequent layer of powder is added. Subsequently, a 3D object is created through the stacking of layers [26]. Liquid-phase materials or slurries containing fine powders, such as ink, are utilized in material jetting to produce droplets that deposit onto the substrate layer-by-layer. The sheet lamination technique uses metal sheets as a feedstock material and a laser or ultrasonic wave as an energy source. By applying mechanical pressure and a localized energy source, the sheets of metal stack together via diffusion, forming a 3D object [26].

Most metallic AM methods involve the heating and melting of feedstock material through a laser beam, followed by rapid solidification. As a result, the microstructure differs from that of as-cast materials with the same composition. The microstructure that is obtained after AM can be classified into two categories: those that solidify in a cellular-dendritic regime and those that form a columnar microstructure. For instance, a columnar microstructure was observed in Ti6Al4V processed by selective laser melting [21,28]. On the other hand, 316L and Inconel 718 alloys that were manufactured by the same method were shown to be solidified in the cellular–dendritic regime [29]. The post-solidification microstructure includes submicron-scale cells of dislocations, which are accumulated due to the thermal stresses caused by the cyclic expansion and contraction of the material during the repetitive heating of the layers in AM processing [30].

Furthermore, AM-processed material typically exhibits a textured microstructure, with grains in cubic materials being preferentially oriented in the <100> directions perpendicular to the substrate. This preference is due to the lower, close-packed density of this direction, which promotes faster growth than other crystallographic directions [21,31,32]. The <110> fiber texture was also observed in some materials, for instance, in 316L stainless steel [33]. Non-equilibrium microstructures such as metastable phases, solute trapping and metallurgical defects (porosity, lack of fusion) are among the distinguishing bulk microstructural features [31,34,35]. AM-produced parts can contain defects and stress-risers, resulting in their containing unfavorable mechanical properties. Consequently, post-processing treatment is necessary to improve the mechanical and functional characteristics of the AM-manufactured components. These post-processing methods can include the coating of the surface, heat treatment, surface roughness improvements and sintering [36]. For instance, the positive influence of heat treatment on the microstructure and wear properties of 316L steel was reported in [37,38]. The heat treatment of Inconel 718 was used to homogenize the microstructure and improve the mechanical properties [29]. Cain et al. showed that annealing improved the fracture toughness and the fatigue crack growth resistance of Ti6Al4V alloy compared to the as-built condition, and additionally reduced the anisotropy of the performance of the material [39].

Among the various steels, 316L austenitic stainless steel is highly favored due to its remarkable corrosion resistance [40,41], and strong mechanical properties [42,43]. Furthermore, 316L steel is suitable for a wide range of applications due to alloying elements like Cr, Ni and Mo. Cr and Mo are responsible for the high corrosion resistance due to the formation of a protective passive layer of Cr_2_O_3_ and MoS. Ni improves hardness and strength, while the ‘L’ stands for the low carbon concentration. 316L steel is used in different applications, including aerospace [44] and prosthetic [45,46] fields, as well as petrochemical [47] and automotive industries [48,49].

The bulk parts of 316L stainless steel can be produced by conventional casting, forging or powder metallurgy [50,51,52]. For instance, a femoral stem made of 316L steel was manufactured by investment casting [53]. However, this steel has poor machinability, which leads to increased defects, in part due to the poor thermal conductivity and high ductility of this material [54]. The above reasons led to extensive research on AM-processed 316L steel components [55]. 316L parts can be produced by various AM methods, such as LPBF, DED and BJ [49,56,57,58]. Depending on the type of AM method, laser power, scan speed and other parameters, different microstructures can be observed. For instance, 316L austenitic stainless steel produced by SLM had a hierarchical microstructure, namely, within each columnar grain, sub-grains/sub-domains with a core-shell morphology were formed. [59]. Columnar grains were also observed in 316L steel produced by the DED method [60]. It was also reported that large columnar grains are formed along the building direction while fine equiaxed grains at the melt pool boundaries are observed from the transverse direction [61]. Furthermore, a variety of crystallographic orientations, ranging from a strong <100> or <110> texture to a more random texture, were observed [62].

In this study, the formation of a nanostructure in AM-processed 316L steel samples is studied. The nanostructuring was carried out by severe plastic deformation (SPD) using the technique of high-pressure torsion (HPT) to examine the significance of the post-AM microstructural refinement process. In addition to the as-built 316L material, other samples, annealed at different temperatures after 3D printing, were also subjected to HPT. The evolution of the microstructure due to SPD was investigated by X-ray line profile analysis (XLPA), which is a very effective and non-destructive testing method for parameters of the microstructure, such as the crystallite size and the density of lattice defects (e.g., dislocations and twin faults). In addition, the change in the crystallographic texture and the hardness during HPT were monitored and correlated with the formation of the nanostructure. The effect of HPT on the microstructural parameters of the AM-processed 316L stainless steel was compared to the same parameters obtained for a bulk counterpart produced by casting.

## 2. Materials and Methods

### 2.1. AM Processing and Subsequent Annealing of 316L Steel Samples

**Figure 1:**
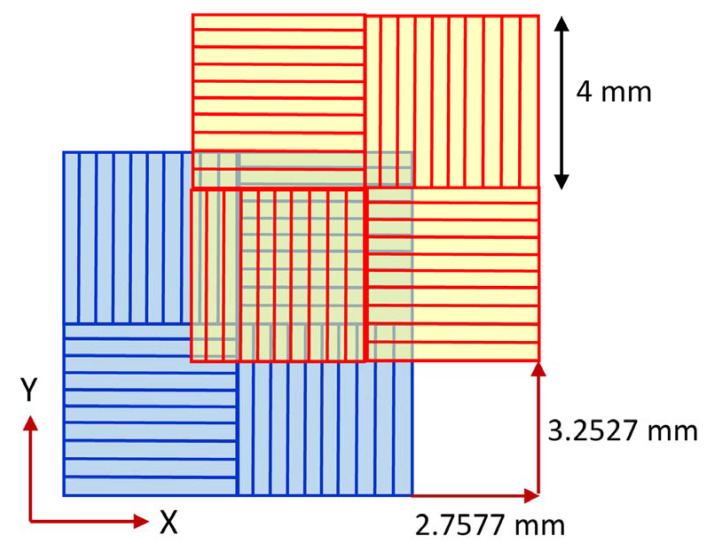
The Chess X–Y scan strategy of 3D printing is used in the present experiments. The blue and red patterns indicate consecutive layers lying perpendicular to the building direction. The lines inside the squares are parallel to the laser scan direction.

Bulk samples were produced by AM from a 316L stainless steel powder with particle sizes of 37 ± 17 μm (manufacturer: Höganäs AB, Höganäs, Sweden) (SS). AM was carried out by laser powder bed fusion (L-PBF) technique using a TruPrint 1000 3D printer (manufacturer: TRUMPF, Ditzingen, Germany). During the printing procedure, the Chess X–Y scan strategy was applied, i.e., the as-printed sample contained a chessboard structure (see Figure 1). This means that square patterns with sides of 4 mm were reprinted by 90°-rotated printing directions and each consecutive layer shifts the pattern by 2.7577 and 3.2527 mm along the *X*- and *Y*-axis, respectively [63,64,65]. The laser power and speed were 113 W and 700 mm/s, respectively. The diameter of the laser spot was 55 μm, while the layer thicknesses and the hatch spacing were 20 and 80 μm, respectively. The parameters of AM processing corresponded to a laser energy density of 161 J/mm^3^. The AM processing was performed in an Ar atmosphere with a gas flow velocity of 2.5 m/s; thus, the oxygen concentration in the 3D printing device was less than 0.3 at.%. Some as-built specimens were heat-treated at 400, 800 and 1100 °C for 30 min, with further cooling in-air.

### 2.2. HPT Processing of the 3D-Printed 316L Steel Samples

The as-built and the subsequently annealed 316L samples were AM-processed in the form of bars with a diameter and length of 12 mm and 65 mm, respectively. Then, disks were cut from the bars with a diameter of 10 mm and a thickness of about 0.8 mm using electrical discharge machining. The as-received disks were processed by HPT under quasi-constrained conditions for ½, 1, 5 and 10 turns at room temperature using a pressure of 6.0 GPa and a rotation speed of 1 rpm.

### 2.3. Phase and Texture Analysis via X-ray Diffraction

The phase content of the AM-processed and the HPT-deformed samples was determined by X-ray diffraction (XRD) using a Smartlab diffractometer equipped with a D/Tex Ultra 250 one-dimensional detector (manufacturer: Rigaku, Tokyo, Japan). In these experiments, CuKα radiation with a wavelength of λ = 0.15418 nm and a Bragg–Brentano (BB) diffraction geometry was utilized in the 2θ range between 40 and 120°. The step size in 2θ was 0.01°. The scanning speed in 2θ was 1.6°/min. In the BB diffraction configuration, a divergent beam was used, with the angle of divergence of 0.2° determined by the incident slit, resulting in a beam width of 3 and 1.2 mm at the beginning and the end of the measurement, respectively (for 2θ of 40 and 120°). The X-ray beam-length-limiting slit was selected as 5 mm. Soller slits of 5° were used in both the incident and the scattered beams. The voltage and current used for the operation of the X-ray tube were 40 kV and 30 mA, respectively. The crystallographic texture was measured by the same XRD apparatus. The texture was characterized by <111>, <200> and <220> pole figures (PFs) obtained using parallel-beam optics. Both the width and the height of the parallel beam were 5 mm due to the applied slits. Soller slits of 0.5° were used in both the incident and the scattered beams. The distance between the X-ray source and the sample, as well as the sample and the detector, was 300 mm for both BB and PF measurements. PFs were plotted using the 3D-Explore software (Ver 3.1.3.0, manufacturer: Rigaku, Tokyo, Japan). The sample surface before XRD experiments was first mechanically polished with 1200, 2500 and 4000 grit SiC abrasive papers, and then the polishing was continued with a colloidal silica suspension (OP-S) with a particle size of 40 nm. Finally, the surface was electropolished at 28 V and 0.5 A using an electrolyte with a composition of 70% ethanol, 20% glycerine and 10% perchloric acid (in vol%).

### 2.4. Characterization of the Microstructure via XLPA

The microstructure of the AM-processed samples and the HPT-processed disks at the center and the edge were characterized using the XLPA method. The surface preparation was the same as described in the previous section. The XRD patterns were measured by a diffractometer operating at 30 kV and 30 mA with CoK_α1_ radiation (wavelength: λ = 0.1789 nm), which was monochromized using a Ge single-crystal monochromator. A parallel X-ray beam with a width and a height of 0.2 and 2 mm, respectively, was used in the experiments. The scattered X-ray radiation was detected by two-dimensional imaging plates. The intensity at a given scattering angle (2θ) was obtained by integrating the signal along the corresponding Debye–Sherrer ring. The 2θ range between 40 and 130° with a step size of 0.015° was measured and evaluated.

The evaluation of the XRD patterns was performed using the Convolutional Multiple Whole Profile (CMWP) fitting method [66]. During CMWP fitting, the diffraction pattern was fitted by the sum of the background spline and the convolution of the instrumental pattern and the theoretical line profiles related to the parameters of the microstructure, namely crystallite size, dislocations and twin faults. For the AM-processed specimens, the instrumental profiles were measured using LaB_6_ standard material. For the HPT-processed samples, the physical broadening of the profiles was much larger than the instrumental broadening; therefore, instrumental correction was not applied in the evaluation of these patterns. The theoretical profile functions related to the crystallite size, dislocations and twin faults are provided in Ref. [67]. The following microstructure parameters, obtained by the CMWP fitting, are presented in this study: the area-weighted mean crystallite size, the average dislocation density and the twin fault probability. The latter quantity corresponds to the fraction of {111} crystallographic planes containing twin faults [67].

### 2.5. Hardness Testing

The microhardness of the as-built and heat-treated samples, as well as the microhardness along the diameter of the HPT-processed disks, was measured using a Zwick Roell ZHµ hardness tester (manufacturer: ZwickRoell LP, Kennesaw, GA, USA). A Vickers indenter loaded with 500 g was used for the measurements, and the dwell time was 10 s. The experiments were performed at room temperature. The statistical error of the hardness values was calculated from 10 individual measurements performed on the AM-processed samples and the edge of the disks processed by HPT.

## 3. Results

### 3.1. Microstructure and Crystallographic Texture of the AM-Processed and Annealed Samples

**Figure 2:**
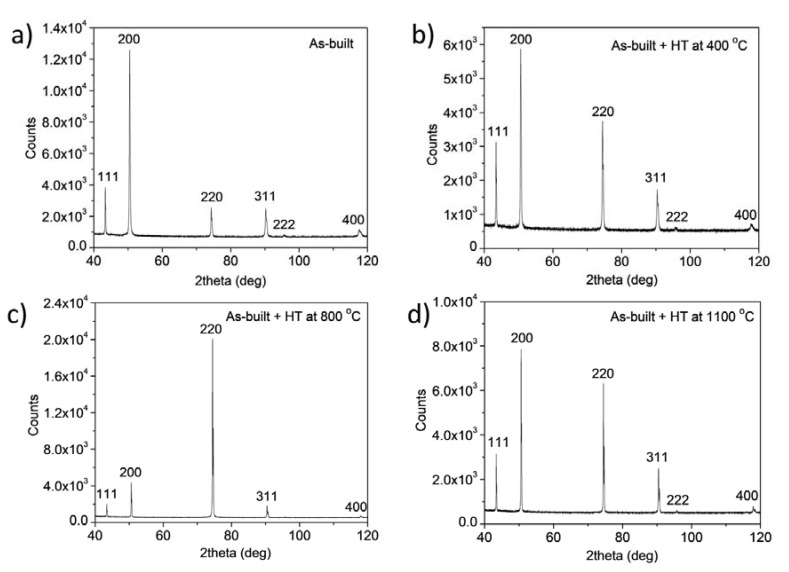
XRD patterns taken on the as-built 316L steel sample (**a**), and the heat-treated specimens (denoted as HT) at 400 (**b**), 800 (**c**) and 1100 °C (**d**) after AM processing.

**Figure 3:**
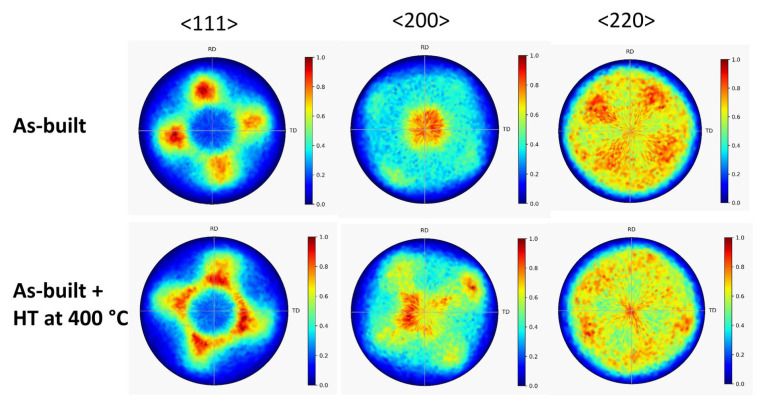
<111>, <200> and <220> XRD pole figures obtained on the as-built 316L steel sample and the specimens that were heat-treated at 400 °C after AM processing (denoted: “As-built + HT at 400 °C”).

**Figure 4:**
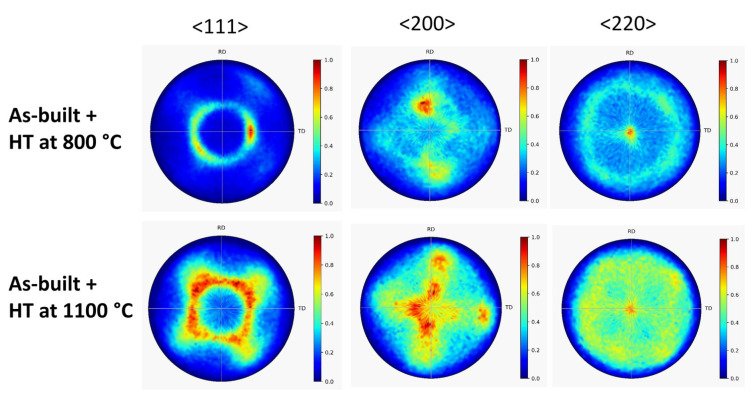
<111>, <200> and <220> XRD pole figures obtained on the specimens heat-treated at 800 and 1100 °C after AM processing (denoted: “As-built + HT at 800 °C” and “As-built + HT at 1100 °C”, respectively).

Figure 2 shows the XRD patterns taken on the as-built 316L steel sample, and the specimens annealed at 400, 800 and 1100 °C after AM processing. The diffractograms suggest that even in the as-built state, and also after the heat treatments, the samples have a full face-centered cubic (fcc) structure without any secondary phase. In addition, the very high intensity of reflections 200 and/or 220 suggests a significant crystallographic texture in the as-built and heat-treated samples. Therefore, <111>, <200> and <220> pole figures were measured by XRD, as shown in Figure 3 and Figure 4. The pole figures confirm <100> texture for the as-built sample while the subsequently annealed specimens have <110> texture.

**Figure 5:**
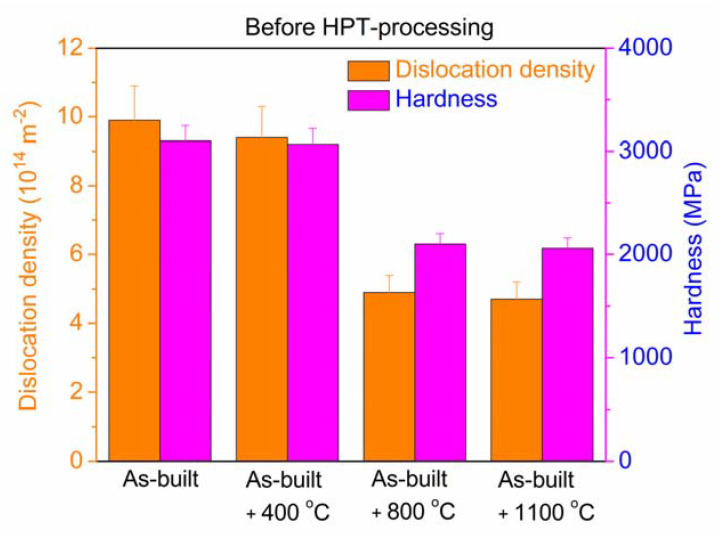
The dislocation density and the hardness of the as-built 316L steel sample and the specimens annealed at 400, 800 and 1100 °C after AM processing.

The dislocation density and the hardness for the as-built and the annealed 316L specimens are shown in Figure 5. The as-built sample contains a high density of dislocations (about 1 × 10^15^ m^−2^), even before HPT deformation. Most probably, these are grown-in dislocations formed during AM processing. The dislocation density did not change considerably during annealing at 400 °C, while the heat treatment at 800 and 1100 °C yielded a significant reduction to about 4–5 × 10^14^ m^−2^. The crystallite size and the twin fault probability were higher and lower, respectively, than the detection limits of the presently applied XLPA method (about 500 nm for the crystallite size and 0.1% for the twin fault probability). The hardness shows a similar trend to the dislocation density, as revealed in Figure 5. Namely, in the as-built state and after annealing at 400 °C, its value is high (about 3000 MPa), which reduced to ~2000 MPa when the temperature of the heat treatment increased to 800 and 1100 °C.

### 3.2. Effet of HPT on the Microstructure and Texture of the AM-Processed and Annealed Specimens

**Figure 6:**
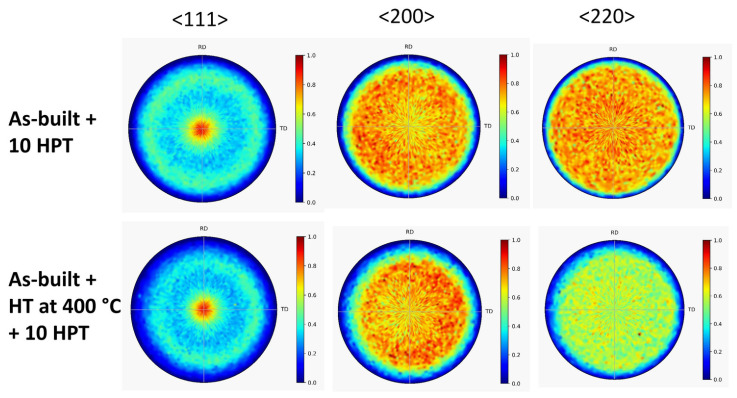
<111>, <200> and <220> XRD pole figures obtained after 10 turns of HPT for the as-built 316L steel sample and the specimen annealed at 400 °C after AM processing (denoted “As-built + 10 HPT” and “As-built + HT at 400 °C + 10 HPT”, respectively).

**Figure 7:**
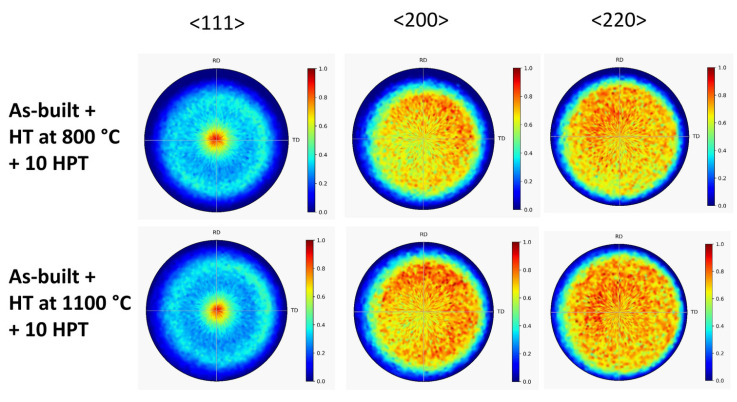
<111>, <200> and <220> XRD pole figures obtained after 10 turns of HPT for the specimens annealed at 800 and 1100 °C after AM processing (denoted “As-built + HT at 800 °C + 10 HPT” and “As-built + HT at 1100 °C + 10 HPT”, respectively).

Figure 6 shows the XRD pole figures obtained after 10 turns of HPT for the as-built 316L steel sample and the specimen annealed at 400 °C after AM processing. It is evident that HPT deformation resulted in the formation of a <111> texture. The same effect was observed for the samples annealed at 800 and 1100 °C after 10 HPT turns, as revealed by the pole figures in Figure 7.

**Figure 8:**
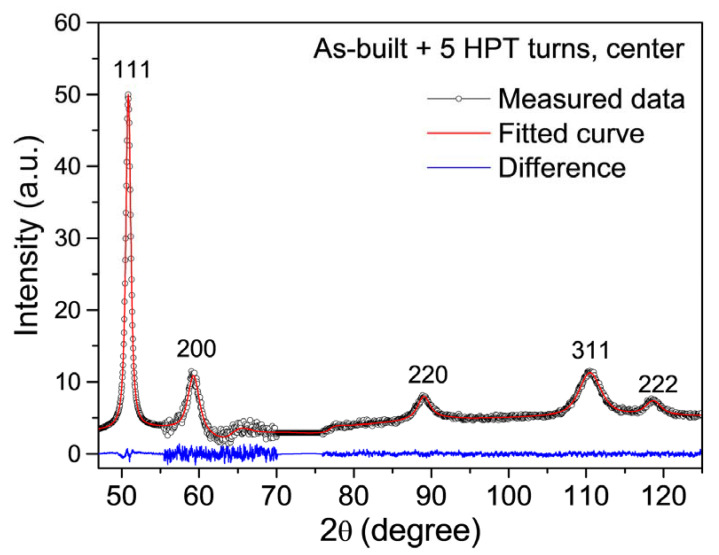
CMWP fitting of the XRD pattern taken in the center of the as-built disk after five turns of HPT. The open circles and the red curve correspond to the measured and calculated diffractograms, respectively. The difference between them is shown by the blue curve.

**Figure 9:**
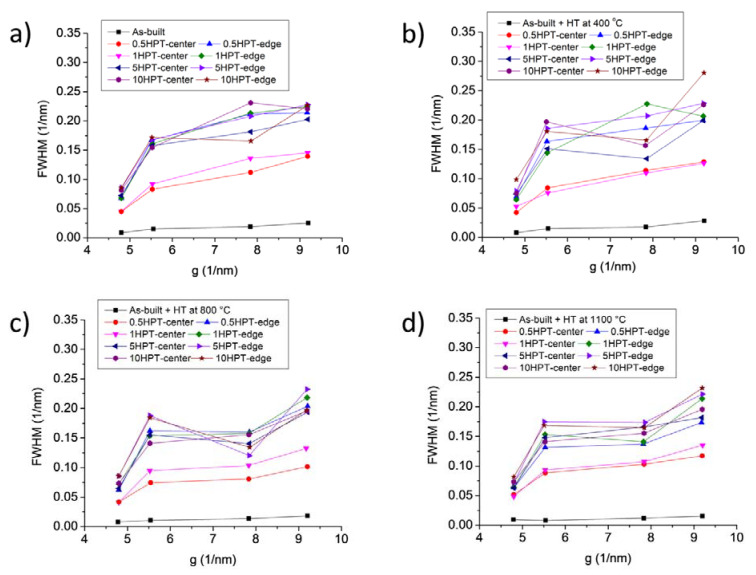
FWHM versus the magnitude of the diffraction vector (g) for the center and the edge of the disks processed by different numbers of HPT turns (Williamson–Hall plot). As-built (**a**); as-built and then heat-treated at 400 (**b**), 800 (**c**) and 1100 °C (**d**).

The microstructure development during HPT processing in the as-built sample and the specimens annealed at 400, 800 and 1100 °C was investigated by XLPA. As an example, Figure 8 shows the XRD pattern taken in the center of the as-built disk after five turns of HPT. The full width at half maximum (FWHM = Δ(2θ)cos θ/λ, where θ is the Bragg angle of the peak, Δ(2θ) is the peak width and λ is the wavelength of X-rays) of the XRD reflections versus the magnitude of the diffraction vector (g = 2sin θ/λ) is plotted in Figure 9 for the center and the edge of the disks processed by different numbers of HPT turns (Williamson–Hall plot). In addition, the Williamson–Hall plots for the samples before HPT are also shown in the figure. In general, the HPT-processed samples exhibited very broad XRD peaks due to the refinement of the crystallite size and the increase in the lattice defect density during SPD. Figure 9 reveals that the difference between the peak breadths of the as-built sample and the specimens annealed at high temperatures (800 and 1100 °C) diminishes when HPT progresses. In addition, except for the center parts of the disks processed for ½ and 1 turn, the data for the HPT-processed samples in the Williamson–Hall plots are close to each other, indicating a saturation of the microstructure parameters. The quantitative characterization of the nanostructure obtained by HPT is presented in the next paragraph.

**Figure 10:**
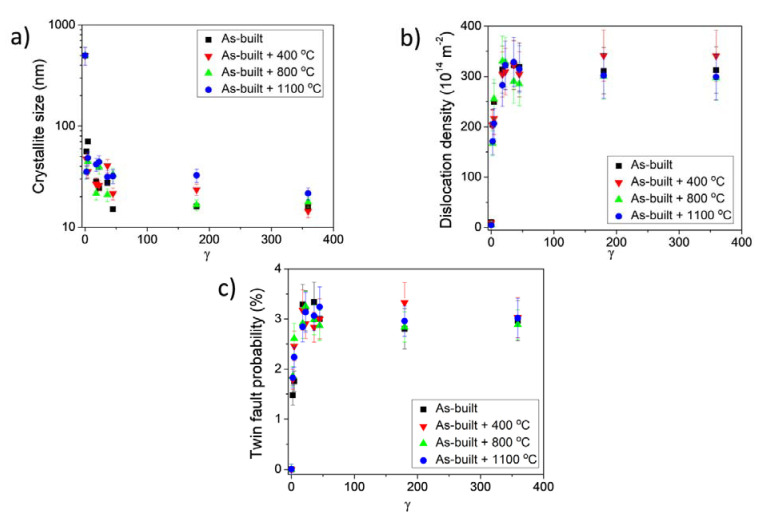
The crystallite size (**a**), the dislocation density (**b**) and the twin fault probability (**c**) versus the shear strain imposed during HPT (*γ*) for the as-built sample and the specimens heat-treated at 400, 800 and 1100 °C after AM-processing.

The crystallite size, the dislocation density and the twin fault probability in the HPT-processed samples were determined by the CMWP fitting of the XRD patterns. As an example, Figure 8 shows the CMWP fitting in the center of the as-built disk deformed by five turns of HPT. The crystallite size, the dislocation density and the twin fault probability obtained by CMWP are shown in Figure 10a,b and 10c, respectively, as a function of the shear strain (γ) that evolved during HPT, which was determined as:

(1) $$\gamma =\frac{2\pi rn}{t},$$

where *n* is the number of HPT turns, *r* is the distance from the disk center and *t* is the thickness of the specimen [27]. This study uses *t* ≈ 0.8 mm for all numbers of turns, and *r* = 0.5 and 4.5 for the center and edge positions, respectively. Based on Equation (1), the lowest and highest shear-strain values in the present study were ~2.2 and ~360, respectively, which are characteristic at the disk center for ½ turn and edge for 10 turns. Figure 10 shows that the crystallite size decreased, while the dislocation density and the twin fault probability increased with increases in the shear strain for all four studied states of the AM-processed 316L steel (as-built and annealed). As mentioned above, the crystallite size before HPT was higher than the detection limit of the presently applied XLPA method (about 500 nm); therefore, this value was plotted in Figure 10a at γ=0. A considerable difference between the evolutions of the microstructural parameters during HPT for the four states cannot be observed. For both the as-built and the annealed materials, the parameters of the microstructure were saturated at the shear strain of about 18. The saturation values of the crystallite size, dislocation density and twin fault probability were about 20 nm, 3 × 10^16^ m^−2^ and 3%, respectively. Thus, it can be concluded that HPT deformation blurred the difference between the initial microstructures of the as-built and annealed 316L steel materials.

### 3.3. Influence of HPT on the Hardness of the AM-Processed and Annealed Samples

**Figure 11:**
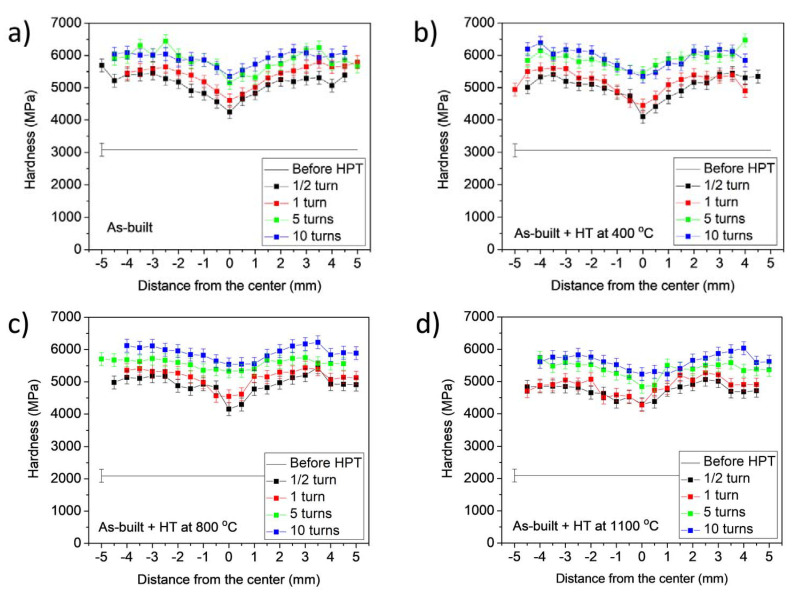
The hardness versus the distance from the center for different numbers of HPT turns in the cases of the as-built 316L material (**a**), and the samples heat-treated at 400 (**b**), 800 (**c**) and 1100 °C (**d**) after AM processing.

**Figure 12:**
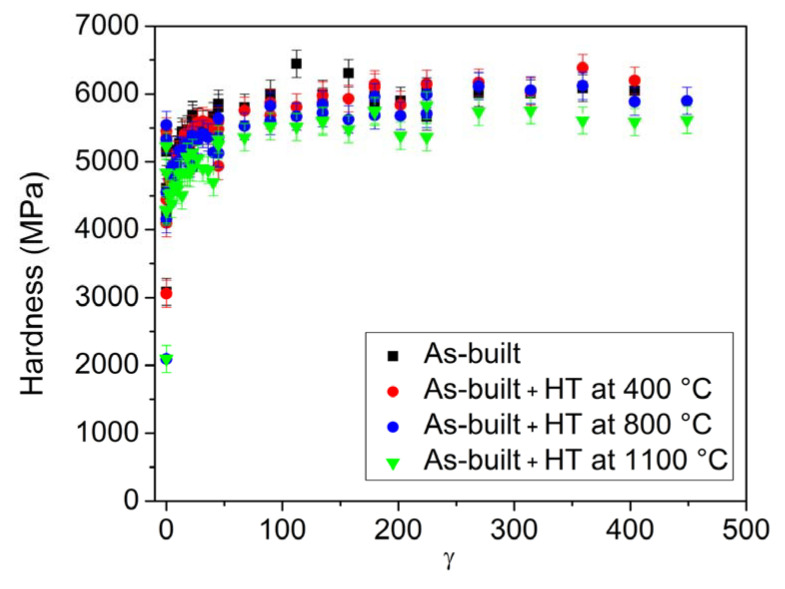
The hardness evolution as a function of the shear strain imposed during HPT (*γ*) for the as-built 316L material and the samples heat-treated (denoted as HT) at 400, 800 and 1100 °C after AM-processing. The data shown in Figure 11 are used here.

Figure 11 shows the hardness as a function of the distance from the center of the disks processed for ½, 1, 5 and 10 turns of HPT. As a reference, the hardness before HPT also indicated for all four states of the AM-processed 316L steel. The hardness increases with increasing distance from the center and the number of turns, as expected. Perfect saturation along the disk radius was not achieved even after 10 turns of HPT, i.e., in the center, the hardness was slightly lower than at the disk edge. The considerable difference between the evolutions of the hardness during HPT for the four initial states (as-built and heat-treated) cannot be observed. This is also confirmed in Figure 12, where the hardness versus the shear strain is plotted using all the values shown in Figure 11. The saturation value of the hardness is about 6000 MPa for all four states of the AM-processed 316L steel. Only the material annealed at 1100 °C after AM processing has a slightly lower maximum hardness; however, this difference is marginal if we consider the uncertainty of the values.

## 4. Discussion

The present investigations revealed a high density of dislocations (about 10^15^ m^−2^) in the as-built 316L steel. These are grown-in defects formed in order to reduce the mismatch stresses between the grains developed under the laser beam during AM processing. The thermal stresses caused by the intensive temperature gradient during 3D-printing may also contribute to the high dislocation density. In as-cast steel counterparts, the dislocation density is at least two orders of magnitude lower, since it is under the detection limit of XLPA which is 10^13^ m^−2^ [67]. It seems that a high dislocation density forms in AM-processed 316L steel samples, irrespective of the laser beam scanning pattern. Indeed, using the same powder and AM conditions, a similar dislocation density was measured when the printing process used sequential line scanning with a 90° rotation of the scan vector between successive layers, instead of the chessboard pattern that is applied at present [68]. Due to the large defect density, the AM-processed 316L steel sample exhibited a much higher hardness than the as-cast counterpart (3000 MPa versus 1300 MPa) [68]. It should be noted that the hardness of the 316L steel made using the AM processing using the chessboard pattern was slightly higher than that for sequential line scanning (about 2500 MPa [68]). This difference can be attributed to the different textures that developed in the two materials processed with different scan strategies. Namely, for sequential line scanning and chessboard patterns, sharp <110> and a less sharp <100> textures formed, respectively. Since the indentation Schmid factor for <110> texture is higher than for <100> texture [69], a lower hardness is expected for the sequential line scanning pattern obtained by the experiments. Former studies have shown that the laser energy density of 3D printing and the relative orientation between the building direction and the axis of loading in mechanical testing also have an influence on the response of AM-processed 316L steels [56,57,59]. The present investigation revealed that heat treatment for 30 min after AM processing at a temperature of 800 °C or higher caused a reduction in the dislocation density and, consequently, the hardness also decreased. A similar reduction in hardness due to heat treatment above 1000 °C was observed in a previous study [57]. On the other hand, at 400 °C, the dislocation density and the hardness remained practically the same as that before annealing, which suggests the good stability of the as-built microstructure of 316L steel at moderate temperatures.

The difference in the laser scanning pattern had no significant effect on the saturation microstructural parameters achieved at high-HPT shear strains. Namely, the minimum crystallite size, the maximum dislocation density and twin fault probability values achieved by HPT were 20–30 nm, 3–3.5 × 10^16^ m^−2^ and 3–4%, respectively, for both chessboard and sequential line scanning patterns. The microstructure was saturated at the shear strain of ~18 for both laser beam scanning patterns. Additionally, the formation of a strong <111> texture during HPT was also similar for the two types of scanning. As a consequence, the saturation hardness values after HPT were close for the chessboard and sequential line scanning patterns (about 6000 and 5200 MPa, respectively). On the other hand, the evolution of the microstructure during HPT in an as-cast 316L steel is very different from that observed for the AM-processed counterparts, as discussed in the next paragraph.

In a former investigation on HPT processing of an as-cast 316L steel, it was found that the phase composition changed during SPD [70]. Namely, the initial fcc structure (γ-phase) was partially transformed to martensitic body-centered cubic (bcc) α- and hexagonal close-packed (hcp) ε-phases. In the saturation state, the HPT-processed as-cast 316L steel contained 70% α-, 25% γ- and 5% ε-phases. The higher stability of the fcc structure in the AM-processed samples can be attributed to the higher Ni content. The chemical composition of the 3D-printed 316L steel samples being studied was 66.2% Fe, 16.5% Cr, 12.1% Ni, 2.4% Mo, 1.5% Mn and 0.6% Si (in wt.%) [64]. On the other hand, the formerly investigated as-cast 316L steel contained 69.1% Fe, 17.2% Cr, 9.0% Ni, 2.1% Mo, 1.0% Mn, 0.8% Si, 0.5% Cu and 0.3% Co (in wt.%) [70]. It is well-known that Ni stabilizes the fcc structure during the deformation of 316L steel. It seems that the critical Ni content for preventing 316L steel from martensitic phase transformation during HPT processing is between 9 and 12 wt.% Ni. It is worth noting that, despite the large difference between the phase compositions of the as-cast and AM-processed 316L steel samples, after HPT, their saturation hardness values were very close (about 6000 MPa). This observation can be explained by the compensation of the effect of the intrinsically harder bcc martensite with a higher defect density in the softer fcc phase. Namely, the saturation dislocation density in the fcc phase for both as-cast and AM-processed 316L steel after HPT was much higher (3–3.5 × 10^16^ m^−2^) than that in the bcc phase (about 1.3 × 10^16^ m^−2^). In addition, the high twin fault probability (3–3.5%) also hardened the HPT-processed fcc phase. The high lattice defect density in fcc 316L steel can be attributed at least partly to the low stacking fault energy (about 20 mJ/m^2^), since this effect causes deformation twinning and a high degree of dislocation dissociation into partials, thereby hindering their annihilation. Therefore, the hardness that is achievable by HPT at room temperature is not considerably influenced by the Ni content of 316L steel.

## 5. Conclusions

In this study, the formation of a nanocrystalline microstructure in AM-processed 316L steel that was severely deformed by the HPT technique was investigated using the XLPA method. In addition, the change in the crystallographic texture due to HPT was revealed. Four initial states were subjected to HPT: an as-built material and its counterparts after being heat-treated after AM at 400, 800 and 1100 °C for 30 min. The hardness evolution during HPT processing was also tested. The following conclusions were drawn from the experimental results:

1. Before HPT, the as-built 316L material contained a high dislocation density of about 10^15^ m^−2^ which is at least two orders of magnitude greater than in an as-cast counterpart. These dislocations in the AM-processed sample are most probably grown-in defects that formed in order to reduce the mismatch stresses between the neighboring grains. Annealing the as-built material at 400 °C did not yield a significant decrease in the dislocation density. However, at 800 and 1100 °C, the dislocation density was reduced to half of the value determined before the heat treatment. Accordingly, the hardness decreased due to annealing at 800 and 1100 °C.
2. HPT resulted in a decrease in the crystallite size from a value higher than 500 nm to about 20 nm at the shear strain of ~18 or higher. Simultaneously, the dislocation density and the twin fault probability increased and reached saturation values of about 3 × 10^16^ m^−2^ and 3%, respectively, at the same shear strain. Due to the reduction in the crystallite size and increase in the defect density, the hardness increased and became saturated with a value of about 6000 MPa. Annealing after AM processing had no considerable effect on the evolution of the microstructure and hardness during HPT. The <100> and <110> crystallographic texture of the as-built and heat-treated samples changed to a <111> preferred orientation during HPT.
3. The AM-processed 316L samples, either before or after annealing, have a higher hardness (2000–3000 MPa) than the as-cast counterpart (1300 MPa) due to the high density of grown-in dislocations. During HPT, the as-cast sample exhibited martensitic phase transformation from fcc to bcc, which was not observed for the 3D-printed samples, due to the stabilization effect of the higher Ni content. On the other hand, the saturation hardness after HPT was similar for the as-cast and AM-processed 316L steel materials, since the hardening effect of the bcc phase in the former sample was compensated with the higher density of lattice defects in the fcc phase of the latter specimen.

## Data Availability

Data are available on request due to restrictions.
